# Supplementary material for: Dietary Isothiocyanates, Sulforaphane and 2-Phenethyl Isothiocyanate, Effectively Impair Vibrio cholerae Virulence
Source: Int J Mol Sci. 2021 Sep 22;22(19):10187. doi: 10.3390/ijms221910187 (PMC8508596; doi:10.3390/ijms221910187)
Supplement: Supplementary file 1 [file ijms-22-10187-s001.zip › ijms-1339802-supplementary.pdf]

International Journal of
Molecular Sciences

MDPI

Supplementary materials

Dietary Isothiocyanates, Sulforaphane and 2-Phenethyl Isothiocyanate, Effectively Impair *Vibrio cholerae* Virulence

Klaudyna Krause[1], Agnieszka Pyrczak-Felczykowska[3], Monika Karczewska[1], Magdalena Narajczyk[4], Anna Herman-Antosiewicz[2], Agnieszka Szalewska Pałasz[1], Dariusz Nowicki[1*]

[1] Department of Bacterial Molecular Genetics, Faculty of Biology, University of Gdansk, Wita Stwosza 59, 80-308, Gdansk, Poland
[2] Department of Medical Biology and Genetics, Faculty of Biology, University of Gdansk, Wita Stwosza 59, 80-308, Gdansk, Poland.
[3] Department of Physiology, Medical University of Gdansk, 80-211 Debinki 1, 80-211, Gdansk, Poland.
[4] Department of Electron Microscopy, Faculty of Biology, University of Gdansk, Wita Stwosza 59, 80-308 Gdansk, Poland
* Correspondence: dariusz.nowicki@ug.edu.pl; Tel.: +48 58 523 60 65

Supplementary data

Table S1. Primer list used in this study

Name	Direction	Tm (°C)	Product size	Sequence	Reference
ctxA	left	64.1	206 bp	TTGTTAGGCACGATGATGGA	This study
	right	63.9		TGGAATCCCACCTAAAGCAG	This study
ctxB	left	63.9	239 bp	GCATATGCACATGGAACACC	This study
	right	63.6		GCAATCCTCAGGGTATCCTTC	This study
tcpA	left	60.1	201 bp	ACGCAAATGCTGCTACACAG	This study
	right	60.0		CCCCTACGCTTGTAACCAAA	This study
toxT	left	59.9	184 bp	TGGGCAGATATTTGTGGTGA	This study
	right	59.4		AAACGCTAGCAAACCCAGAC	This study
toxR	left	66.9	65 bp	CGAAACGCGGTTACCAATTG	This study
	right	67.1		GCTTTCGCGAGCCATCTCT	This study
toxS	left	65.1	72 bp	TGCCATTAGGCAGATATTTCACA	This study
	right	67.3		GCAACCGCCCGGCTAT	This study
tcpP	left	59.9	154 bp	TGAGTGGGGGAAGATAAACG	This study
	right	60.2		CCCCGGTAACCTTGCTAAAT	This study
tcpH	left	60.4	181 bp	GACCGATCCACAAGGTAACG	This study
	right	59.4		CTTGTAAGGGAAGGCGAGAA	This study
hns	left	60.0	182 bp	GCACGCGAACTGACTATTGA	This study
	right	60.3		GCAGAAATAAGCGCTTCGAG	This study
recA	left	64.7	71 bp	CAATTTGGTAAAGGCTCCATCAT	This study

	right	65.8		CCGGTCGAAATGGTTTCTACA	This study
acfB	left	60.1	199 bp	TCCAAGGTCGATGTGTCAAA	This study
	right	60.0		TTGGATCGAATCCTGTAGGC	This study
tcpI	left	59.7	217 bp	GGCCTGAGACCAGATTTTTG	This study
	right	60.0		CTCACTATTGCCACCCCTGT	This study

1. SFN and PEITC anti-biofilm activity

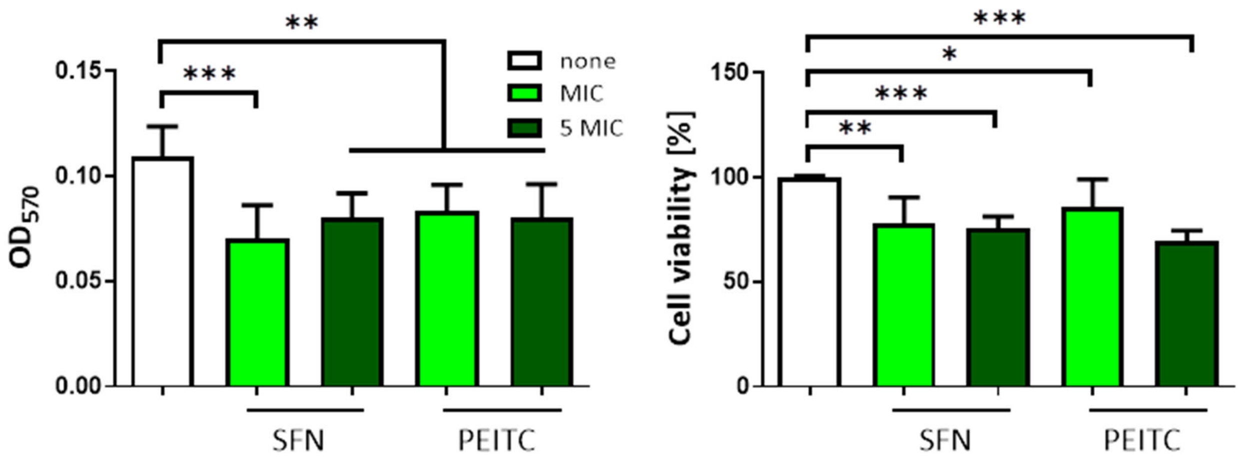

(a) (b)

Figure S1. SFN and PEITC prevent biofilm formation. (a) ITC impact on biofilm mass assessed in crystal violet assay. (b) The metabolic activity of SFN and PEITC treated cells assessed in MTT assay. The ITC suspension (20µl) in MH broth with concentrations as indicate was added to the culture at starting point together with inoculum (180µl, overnight culture). The experiment was repeated independently three times. Data are shown as means +/-1 SD. The significance of differences between the results and the control was tested using the Student's t-test. Statistical significance is marked with asterisks: *** - p <0.001; ** - p <0.01; * - p <0.05.

2. Galleria mellonella surrogate host of V. cholerae infection

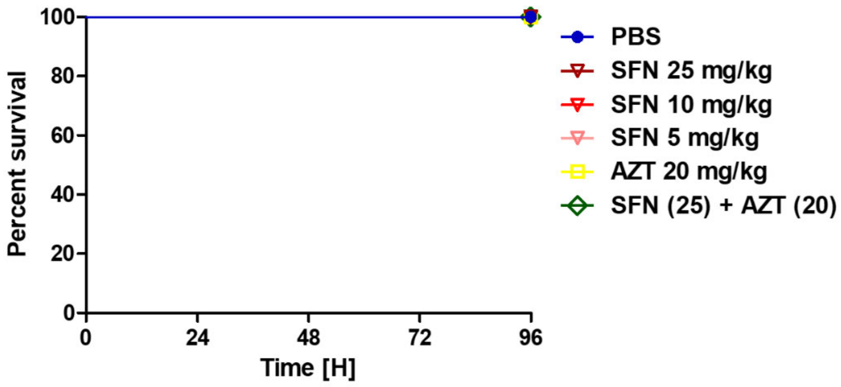

Figure S2. Kaplan-Meier survival analysis of antimicrobia compounds toxicity assessed on Galleria mellonella larvae. Larvae (n=15) were injected with (10µl) of PBS (blue circle) or SFN at concentarion range of 5 to 25 mg/kg (red triangle) and AZT 20 mg/kg (yellow scuare), or SFN (25mg/kg) and AZT (20 mg/kg) in mixture (green deltoid).The experiment was repeated independently three times. Surface sterilization of insects with EtOH was performed prior to each injection. The larvae were then incubated at 37 °C, and survival and melanisation were recorded in the next 96h. Larvae were scored as dead when they ceased moving, changed from their normal pale cream coloration to brown, and failed to respond when gently manipulated with a pipette tip. Survival 96h post-infection was recorded.
